# Association of Empirical Dietary Atherogenic Indices with All-Cause and Cause-Specific Mortality in a Multi-Ethnic Adult Population of the United States

**DOI:** 10.3390/nu11102323

**Published:** 2019-10-01

**Authors:** Mohsen Mazidi, Niki Katsiki, Dimitri P. Mikhailidis, Marcin A. Bartłomiejczyk, Maciej Banach

**Affiliations:** 1 International College, University of Chinese Academy of Science (IC-UCAS), Beijing 100101, China; 2 First Department of Internal Medicine, Division of Endocrinology and Metabolism, Diabetes Center, AHEPA University Hospital, Aristotle University of Thessaloniki, PO 546 21Thessaloniki, Greece; nikikatsiki@hotmail.com; 3 Department of Clinical Biochemistry, Royal Free Campus, University College London Medical School, University College London (UCL), NW3 2QG London, UK; mikhailidis@aol.com; 4 Department of Hypertension, Chair of Nephrology and Hypertension, Medical University of Lodz, 93-338 Lodz, Poland; marcin.bartlomiejczyk@umed.lodz.pl (M.A.B.); maciejbanach@aol.co.uk (M.B.); 5 Polish Mother’s Memorial Hospital Research Institute (PMMHRI), 93-338 Lodz, Poland; 6 Cardiovascular Research Centre, University of Zielona Gora, 65-046 Zielona Gora, Poland

**Keywords:** serum uric acid, apolipoprotein B, mortality, cardiovascular disease, cancer, dietary uricaemia score, dietary atherogenic score

## Abstract

Serum uric acid (SUA) and apolipoprotein B (apoB) are markers of the risk of morbidity and mortality. However, no study has investigated their role, simultaneously with nutritional factors, on the risk of mortality. We calculated the dietary uricaemia score (DUS) and the dietary atherogenic score (DAS) and evaluated their associations with the risk of all-cause and cause-specific mortality. Data from the NHANES 1999–2010 study were used. Vital status through the 31 December 2011 was ascertained. Reduced rank regression models followed by stepwise linear regression analyses were applied on 39 macro/micronutrients to identify a dietary pattern most predictive of SUA (DUS) and apoB (DAS). Overall, 20,256 participants were included (mean age: 47.5 years; 48.7% men). DUS consists of 14 contributors (eight positive, six negative), whereas DAS consists of 23 contributors (six positive, 17 negative). An increasing risk of cause-specific mortality was found across the quartiles (Q) of DUS, i.e., participants with the highest score of DUS (Q4) had a greater risk of all-cause (hazard ratio (HR): 1.17, 95% confidence interval (CI): 1.07–1.30), cardiovascular disease (CVD) (HR: 1.36, 95%CI: 1.21–1.59) and cancer (HR: 1.06, 95%CI: 1.01–1.14) mortality compared with Q1. Similarly, participants at the highest DAS quartile had 25, 40 and 11% greater risk of all-cause, CVD and cancer mortality, respectively, compared with Q1. For the first time, we reported an underlying shared link between two atherosclerosis factors (SUA and apoB) and nutrients, as well as their joint adverse impact on all-cause and cause-specific mortality.

## 1. Introduction

Non-communicable diseases (NCDs), mainly cardiovascular disease (CVD), stroke, cancer, diabetes and chronic respiratory disease, are an increasing cause of morbidity and mortality worldwide [1]. Worldwide, regardless of gender, age or membership of social group, the spread of NCDs is a global crisis [2]. According to the World Health Organization (WHO), 71% of deaths worldwide are caused by NCDs annually (approximately 41 million) of which >43% (17.9 million people) are caused by CVD. It is worth adding that, according to the WHO, nine million people die from cancer every year, respiratory diseases contribute 3.9 million deaths, and diabetes 1.6 million deaths annually [3].

Dietary risk factors contribute to mortality and morbidity from NCDs [4]; food consumption in developed countries is dominated by unhealthy diets [2]. In contrast, in 2017, in papers authored by Jannasch et al. and Perez-Martinez et al., have shown that the Mediterranean diet reduces the risk of type 2 diabetes, cancer incidence, as well as CVD [5,6].

In this context, the Prevención con Dieta Mediterránea trial (PREDIMED) demonstrated the significant role of diet in decreasing CVD events and improving peripheral artery disease (PAD), diabetes mellitus type 2 (T2DM) and breast cancer prevalence [7,8,9]. Similarly, the DASH (Dietary Approaches to Stop Hypertension trial) diet is related to lower risks of CVD events, cancer and T2DM [10]. This evidence emphasizes the role of diet on different NCDs.

The final product of purine metabolism, serum uric acid (SUA), contributes to the proliferation of smooth muscle cells, inflammation and endothelial dysfunction [11,12]. Clinical investigations suggested that SUA may take part in the etiology of atherosclerosis and chronic heart diseases and that it may be a potential marker for CVD risk [13].

According to published meta-analyses of observational studies there may be a relationship between SUA and the risk of heart failure, atrial fibrillation and cancer. SUA could also be an independent predictor of CVD mortality [14,15,16]. Diet can affect SUA levels [17,18]. For example, a randomized clinical trial reported that the DASH diet (based on the consumption of lean meat, poultry and fish, non/low-fat dairy products, vegetables, fruits, fiber, beans and nuts) decreased SUA; this effect was more evident in participants with hyperuricaemia [17,18]. In contrast, a randomized trial of African American adults with controlled hypertension showed that compared with the control group there was no improvement in SUA concentration after partial switching to the DASH diet [19].

Based on the Global Burden of Disease Study it was assumed that the main factor of atherosclerotic CVD is lipoproteins containing apolipoprotein B (apoB) [20]. ApoB is present in cholesterol and triglyceride carrying lipoproteins [21]. A few studies evaluated the link between diet and apoB [22]. Dietary patterns (DP) characterized by higher intake of seafood, low-fat dairy products, fruits, fish, vegetables, and whole cereal have been related to reduced plasma apoB concentrations [23]. However, apoB levels remained unchanged after both a high-carbohydrate meal (consisting of 120 g carbohydrates (~60% sugars) and 8 g fat) and a high-fat meal (consisting of 54 g carbohydrates (~15% sugars) and 28 g fat, containing slightly less (~20%) total calories) [24]. Therefore, there are contradictory findings regarding the effects of DP on apoB.

More indicative predictors of NCDs might be markers that take into account various dietary factors or DP taking into account the complex interactions between nutrients [25] compared with evaluations of just one nutrient [26,27,28]. In this context, we applied DP analysis i.e., statistical methods used to examine the pattern of intake of multiple foods or nutrients and derive single-exposure variables [26,27,29,30]. This approach could facilitate the development of public health recommendations that are more convenient to follow [31]. The aim of our study was to evaluate the role of SUA and apoB together with nutrient parameters on the risk of all-cause and cause-specific (CVD and cancer) mortality. We have chosen SUA and apoB because it have previously reported that the levels of these two CVD risk factors are affected by the diet [32,33,34,35].

## 2. Materials and Methods

### 2.1. Population

The US National Health and Nutrition Examination Survey (NHANES) database was analysed for this study [36]. All participants signed written informed consent approved by The National Center for Health Statistics (NCHS) Research Ethics Review Board. Presented results were prepared according to the analysis of data for 2-year NHANES survey cycles between 1999 and 2010, restricted to participants aged ≥18 years. Details on the NHANES Laboratory/Medical Technologists Procedures and Anthropometry Procedures are described elsewhere [37,38].

A blood specimen was drawn from an antecubital vein. Glycated haemoglobin (HbA_1c_) and fasting blood glucose (FBG) were measured using a Tosoh A1C 2.2 Plus Glycohemoglobin Analyzer (Tosoh Bioscience, San Francisco, CA, USA) and a hexokinase method using a Roche/Hitachi 911 Analyzer and Roche Modular P Chemistry Analyzer (Mannheim, Germany), respectively [39]. Other laboratory test details are available in the NHANES Laboratory/Medical Technologists Procedures Manual [40]. Hypertension (HTN) was diagnosed in individuals with systolic blood pressure (SBP) ≥140 mmHg and/or diastolic blood pressure (DBP) ≥90 mmHg, or those on antihypertensive medication [41]. T2DM was defined as a self-reported treatment of diabetes or FBG ≥126 mg/dL [42]. Smoking status was self-reported.

Dietary intake was assessed via 24 h recalls obtained by a trained interviewer, with the use of a computer-assisted dietary interview system with standardized probes, i.e., the United States Department of Agriculture Automated Multiple-Pass Method (AMPM); this method enhances the accuracy and completeness of data collection, while minimizing respondent burden [43,44].

### 2.2. Calculation of the Dietary Hyperuricaemia Score (DUS) and Dietary Atherogenic Score (DAS)

The derivation approach, which we applied was empirical hypothesis-oriented. We used 37 micro and macronutrients: protein (g), carbohydrate (g), total sugars (g), dietary fibre (g), total fat (g), total saturated fatty acids (g), total monounsaturated fatty acids (g), total polyunsaturated fatty acids (g), cholesterol (mg), vitamin E (mg), retinol (μg), vitamin A (μg), α-carotene (μg), β-carotene (μg), β-cryptoxanthin (μg), lycopene (μg), lutein and zeaxanthin (μg), thiamine (vitamin B1) (mg), riboflavin (vitamin B2) (mg), niacin (mg), vitamin B6 (mg), folic acid (μg), choline (mg), vitamin B12 (μg), vitamin C (mg), vitamin K (μg), calcium (mg), phosphorus (mg), magnesium (mg), iron (mg), zinc (mg), copper (mg), sodium (mg), potassium (mg), selenium (μg), caffeine (mg), theobromine (mg), and alcohol (g), and applied them in reduced rank regression (RRR) models to derive a DP predictive of SUA and apoB (as predictors) separately. Then, we used the first factor obtained by RRR and performed further data reduction in stepwise linear regression analyses, with RRR DP as the dependent variable and the 39 micro/macronutrients as independent variables. For weighting, we used regression coefficients. The stepwise linear regression analyses identified 14 micro/macronutrients for DUS and 23 micro/macronutrients for DAS; micro/macronutrients were weighted and summed for each participant to constitute the DUS and DAS. For both DUS and DAS, higher (more positive) scores indicated a higher adherence to uricaemia and atherogenic diets, respectively, whereas lower (more negative) scores indicated lower adherence to uricaemia and atherogenic diets, respectively. We applied the same approach for other CVD risk factors (Under-review).

### 2.3. Mortality

The anonymized data of NHANES 1999–2010 participants were linked to longitudinal Medicare and mortality data using the NHANES assigned sequence number. Mortality follow-up data are available from the date of survey participation until 31 December 2011. We recorded all-cause mortality, as well as mortality due to CVD (I00-I09, I11, I13, I20-I51 and I60-I69) and cancer (C00-C97). The cause of death was determined using the 10th revision of the International Classification of Diseases (ICD-10).

### 2.4. Statistical Analysis

Analyses were conducted according to the guidelines set by the Centers for Disease Control and Prevention for analysis of the NHANES dataset, accounting for the masked variance and using their suggested weighting methodology [45]. Continuous and categorical demographic variables were compared across DUS and DAS quartiles using analysis of variance (ANOVA) and chi-square tests, respectively. Multivariable Cox proportional hazards were applied to determine the hazard ratios (HRs) and 95% confidence intervals (CIs) of mortality (all-cause, CVD and cancer) for DUS and DAS; the first quartile (Q1) was always used as reference. To derive the HR and 95% CIs, we used two different models: Model 1 adjusted for age, gender, race, education, marital status, poverty to income ratio, total energy intake, physical activity, smoking and alcohol consumption; Model 2 adjusted for age, gender, race, education, marital status, poverty to income ratio, total energy intake, physical activity, smoking, alcohol consumption, body mass index (BMI), HTN and T2DM.

A two-sided *p* < 0.05 was used to characterize significant results. Data were analysed using SPSS^®^ complex sample module version 22.0 (IBM Corp, Armonk, NY, USA).

## 3. Results

A total of 20,256 volunteers participated in the study (mean age = 47.5 years; 48.7% men). Their demographic characteristics across the quartiles of DUS and DAS are shown in Table 1. With regard to DAS, participants in the highest quartile (Q4) were younger, had higher a BMI, lower education level, and were more likely to be smokers compared with those in the first quartile (Q1, *p* < 0.001 for all comparisons). Participants in the highest quartiles of DUS and DAS had significantly greater total energy intake as well as greater consumption of total fat and carbohydrates with a lower intake of fibre (*p* < 0.001 for all comparison, Table 1).

Of the 37 micro/macronutrients, 14 were significant contributors to the DUS, with eight positively and six negatively associated. Furthermore, 23 of them were significantly associated with the DAS, six positively and 17 negatively. Common nutrients for both DUS and DAS were: carbohydrate, total fat, saturated fatty acid, cholesterol, sodium and alcohol (positive associations) as well as dietary fibre, polyunsaturated fatty acid, vitamin E, thiamine, niacin, riboflavin vitamin B6, folic acid, lycopene, choline, vitamin C, calcium, phosphorus, magnesium, selenium, potassium and zinc (inverse associations).

During the median follow-up of 11.3 years, 3433 deaths were recorded, including 962 CVD deaths and 799 due to cancer. Risk of death across (cause-specific and all-cause) across the DUS and DAS quartiles were calculated with the use of the multivariable Cox regression model are shown in Table 2. In Model 1, all-cause mortality was increased across the quartiles of DUS; participants in Q3 and Q4 had a 12 and 30% higher risk for all-cause mortality compared with those in Q1 [HR: 1.12 (95%CI: 1.08–1.19) and 1.30 (95%CI: 1.12–1.45) for Q3 and Q4, respectively; *p*-trend = 0.029). After further corrections for BMI, HTN and T2DM in Model 2, the magnitude of the association decreased, but remained significant and positive, for Q3 (HR: 1.09, 95%CI: 1.05–1.15) and Q4 (HR: 1.17, 95%CI: 1.07–1.30; *p*-trend = 0.034). CVD mortality also increased across quartiles of DUS [in Model 1: HR: 1.11 (95%CI: 1.04–1.17) for Q2, 1.16 (95%CI: 1.12–1.20) for Q3 and 1.45 (95%CI: 1.12–1.89) for Q4; *p*-trend < 0.001, Table 2]. After further adjustments in Model 2, the associations were reduced but still significant for Q3 and Q4 [HR: 1.04 (95%CI: 1.02–1.07) and 1.36 (95%CI: 1.21–1.59), respectively; *p*-trend < 0.001]. With regard to cancer mortality, a significant and positive link was observed only for the participants in Q4 of DUS compared with those in Q1 [Model 1: HR: 1.11 (95%CI: 1.04–1.18); Model 2: HR: 1.06 (95%CI: 1.01–1.14)].

Similar results were found for the DAS. In Model 1, participants in Q3 and Q4 had a 30% and 40% higher risk of all-cause mortality compared with those in Q1 (*p*-trend = 0.018); the corresponding values were 9% and 25%, respectively, in Model 2 (*p*-trend = 0.042, Table 2). CVD mortality increased across quartiles of DAS [in Model 1: HR: 1.16 (95%CI: 1.05–1.28) for Q2, 1.34 (95%CI: 1.19–1.55) for Q3 and 1.55 (95%CI: 1.45–1.62) for Q4; *p*-trend < 0.001, Table 2]. After further adjustments in Model 2, the associations were attenuated but still significant [HR: 1.08 (95%CI: 1.02–1.13), 1.28 (95%CI: 1.05–1.54) and 1.40 (95%CI: 1.10–1.79), respectively; *p*-trend < 0.001]. With regard to cancer mortality, a significant and positive link was found only for the participants in Q4 of DAS compared with those in Q1 [Model 1: HR: 1.15 (95%CI: 1.08–1.17, *p*-trend = 0.468); Model 2: HR: 1.11 (95%CI: 1.04–1.19, *p*-trend = 0.327)] (Table 2).

## 4. Discussion

Using data from the NHANES database, we prospectively evaluated the associations of two empirical hypothesis-oriented dietary indices (i.e., the DUS and DAS) that represent diets with increased uricaemia and atherogenic risk, with all-cause/cause-specific mortality. Participants with higher DUS and DAS were more susceptible to all-cause and CVD mortality (all *p* < 0.042). Of note, after correction for cardiometabolic risk factors (i.e., BMI, HTN and T2DM), the associations were diluted but remained significant. Regarding cancer mortality, significant and positive links were observed only between participants in Q4, compared with those in Q1, for both DUS and DAS; the relationships were attenuated after adjustments for BMI, HTN and T2DM. To the best of our knowledge, this is the first study to evaluate the complex effects of adherence to “uricaemia” and “atherogenic” diets on the risk of all-cause and cause-specific mortality.

To date, only a few studies and meta-analyses confirmed that elevated SUA levels increase the risk of heart failure and ischaemic heart disease (IHD) [13], as well as the components of metabolic syndrome [46]. However, the results of the Mendelian randomization study published in 2016 indicated no relationship between SUA and CHD. This suggests that SUA is not a causal factor, but may act as a marker of the development of CVD [47]. The NHANES I Follow-Up Study (n = 5926 individuals, follow-up = 16.4 years) reported that levels of elevated SUA were significantly related to a greater risk of CVD mortality [48]. Furthermore, Zhao et al. in 2013 noted the gender difference (stronger in women than men) between CVD and SUA [16]. Uric acid impairs nitric oxide synthesis (NOS), leading to vascular endothelial dysfunction [49]. In this context, flow-mediated vasodilation, a marker of endothelial function, was significantly lower in hyperuricaemic patients [50]. Of note, endothelial dysfunction represents a pro-thrombotic, pro-constrictive and pro-inflammatory state.

Several dietary factors may affect SUA levels, including (purine-rich) meat and sea-food, alcoholic beverages (especially beer) and sugar-sweetened soft drinks (as SUA raising components), and dairy and coffee as potentially SUA-lowering agents [32]. These dietary factors may influence SUA by providing purines as precursors of uric acid, increasing or decreasing nucleotide turnover, or by affecting the renal excretion of uric acid. In line with our findings, clinical trials reported that subjects with higher adherence to the DASH and Mediterranean diets (rich in polyunsaturated fatty acids, vitamins and minerals) might have lower SUA levels [17,51]. The ATTICA study, comprising 2380 men and women without renal disease or CVD, reported that adherence to the Mediterranean diet was related to lower SUA levels [51].

A cohort study between 2002 and 2012 conducted on a group of 375,163 South Koreans, reported that both low and high SUA concentrations were predictive of increased mortality, supporting a U-shaped association [52]. Although the mechanisms underlying the increased risk of mortality related to low SUA levels is not completely identified, there are some probable explanations. For example, reduced SUA concentrations may reflect malnutrition [53]. Furthermore, uric acid acts as an antioxidant, by increasing superoxide dismutation to hydrogen peroxide, thus decreasing the availability of superoxide and its harmful interaction with nitric oxide [36]. Therefore, low SUA levels may represent a decreased antioxidant capacity. On the other hand, it has been proposed that individuals with hyperuricaemia are at an increased risk of atherosclerotic disease [54].

In accordance with our findings, the Mediterranean diet (diet high in fibres, legumes, fruits, vegetables and unprocessed grains with low consumption of meat and meat products) was reported to increase the size of low-density lipoprotein (LDL) and reduce LDL-apoB100 concentrations, mainly by enhancing LDL catabolism, even in the absence of weight loss in men with metabolic syndrome [55]. Furthermore, a cross-sectional study of 35 pregnant women showed that mothers at the low Mediterranean diet score (representing a diet poor in fibre and folate, rich in cholesterol, with low polyunsaturated (PUFA) + monounsaturated/saturated fatty acids (MUFA/SFA) ratio, and high SFA/carbohydrates (SFA/CHO) and ω-6/ω-3 PUFA ratios) delivered neonates with high LDL-cholesterol, apo B levels and a high apo A1/apo B ratio [56]. Therefore, a healthy diet can play a significant role in prevention of chronic diseases even from an early age [56]. In contrast, a randomized clinical trial comprising 155 T2DM patients indicated no significant changes in apo B levels after 6 months of high fiber or low glycaemic index diet [57]. However, larger studies with a longer follow-up are needed to extract safe conclusions.

### Strengths and Limitations

Major strengths of our study include the design and use of novel, prospective, food-based dietary indexes. Furthermore, analyses were adjusted for a wide range of available covariates, thus reducing the potential for residual confounding bias. With regard to limitations, the analysis relied on a one-time measurement for dietary and covariate data; this may increase within-person variation. Some methodological issues to be considered in interpreting our findings also include potential measurement error in the self-reported dietary and lifestyle data. Furthermore, although we adjusted for several potential variables, we cannot completely rule out confounding by unmeasured factors. Further, in this study because of the high chance of the co-linearity, we were not able to include estimated glomerular filtration rate (eGFR) in our model once we were considered the SUA as a risk factor. However, it would be good for future studies to consider this fact if their model allows them.

## 5. Conclusions

All-cause and CVD mortality significantly increased across quartiles of both DUS and DAS in a large multi-ethnic US adult population. With regard to cancer mortality, significant and positive links were observed only between participants in the highest DUS and DAS quartiles compared with those in the first quartile. These associations were attenuated (but remained significant) after adjustments for BMI, HTN and T2DM. Therefore, dietary interventions to minimize the adverse effect of uricaemia and atherogenic potential of diet might contribute in reducing all-cause and cause-specific mortality. Further research is required to establish the implications of such health policies.

## Figures and Tables

**Table 1:** Characteristics of the study participants based on the Dietary Uricaemia Score (DUS) and the Dietary Atherogenic Score (DAS) quartiles (Q).

Variables		Dietary Uricaemia Score				*p*	Dietary Atherogenic Score				*p*
		Q1 (*n* = 5064)	Q2 (*n* = 5063)	Q3 (*n* = 5605)	Q4 (*n* = 5604)		Q1 (*n* = 5062)	Q2 (*n* = 5065)	Q3 (*n* = 5064)	Q4 (*n* = 5064)	
**Age (Years)**		49.8 ± 0.1	50.3 ± 0.1	46.9 ± 0.2	47.1 ± 0.2	<0.001	51.3 ± 0.1	50.8 ± 0.1	46.1 ± 0.1	47.2 ± 0.1	<0.001
**Gender**	**Men (%)**	39.6	42.8	47.1	56.2	<0.001	41.9	42.6	48.7	53.8	<0.001
	**Women (%)**	60.4	57.2	52.9	43.8		58.1	57.4	51.3	52.3	
**Race/Ethnicity**	**Mexican-American (%)**	23.2	25.8	20.7	28.7	<0.001	19.3	20.8	22.5	22.0	<0.001
	**Non-Hispanic White (%)**	51.8	49.6	50.0	42.1		51.4	49.6	54.1	52.8	
	**Non-Hispanic Black (%)**	17.8	16.8	20.9	17.9		20.6	17.3	20.1	19.8	
**Education Status**	**Less Than High School (%)**	9.6	15.8	19.8	23.1	<0.001	11.9	12.3	11.8	14.8	<0.001
	**Completed High School (%)**	32.4	40.4	41.8	40.9		30.8	36.9	40.6	42.0	
	**More Than High School (%)**	42.4	39.8	38.4	36.9		41.8	41.6	36.5	38.1	
**Smoking (%)**		19.6	22.5	22.0	21.9	<0.001	20.1	19.9	21.8	22.2	<0.001
**Poverty to Income Ratio (n)**		2.5 ± 0.03	2.4 ± 0.02	2.7 ± 0.02	2.5 ± 0.02	<0.001	2.4 ± 0.02	2.5 ± 0.02	2.6 ± 0.03	2.4 ± 0.01	<0.001
**Body Mass Index (kg/m^2^)**		25.4 ± 0.1	26.2 ± 0.08	24.9 ± 01	27.8 ± 0.09	<0.001	24.9 ± 0.1	27.5 ± 0.08	25.3 ± 01	26.8 ± 0.09	<0.001
**Systolic Blood Pressure**		121.3 ± 0.4	118.3 ± 0.4	125.1 ± 0.3	120.1 ± 0.9	<0.001	125.1 ± 0.3	120.3 ± 0.4	122.3 ± 0.1	119.2 ± 0.9	<0.001
**Carbohydrate (g/day)**		198.2 ± 1.9	224.1 ± 2.2	274.2 ± 2.4	323.5 ± 2.1	<0.001	201.3 ± 2.0	224.1 ± 2.1	289.3 ± 2.0	342.6 ± 2.2	<0.001
**Dietary Fiber (g/day)**		20.1 ± 0.1	18.6 ± 0.2	17.2 ± 0.1	14.9 ± 0.1	<0.001	19.8 ± 0.1	19.0 ± 0.1	16.8 ± 0.1	15.4 ± 0.1	<0.001
**Total Fat (g/d)**		58.7 ± 0.3	63.9 ± 0.3	78.4 ± 0.3	100.5 ± 0.3	<0.001	60.4 ± 0.3	65.9 ± 0.3	82.4 ± 0.4	101.4 ± 0.4	<0.001
**Total Energy Intake (kcal/day)**		1682.1 ± 20.4	2004.9 ± 19.5	2298.5 ± 20.1	2841.3 ± 19.8	<0.001	1741.1 ± 20.4	1985.6 ± 19.5	2362.8 ± 20.1	2726.5 ± 22.1	<0.001
**Alcohol Consumption (g/day)**		9.8 ± 0.2	12.3 ± 0.2	11.4 ± 0.2	12.8 ± 0.2	<0.001	12.3 ± 0.2	9.6 ± 0.2	13.0 ± 0.2	11.2 ± 0.2	<0.001

Analysis of variance or chi-square were used to compare the groups across the quartiles. Values expressed as mean ± standard error of mean or %.

**Table 2:** Multivariable-adjusted hazard ratios (95% confidence intervals) for mortality across the Dietary Uricaemia Score (DUS) and the Dietary Atherogenic Score (DAS) quartiles (Q).

		Dietary Uricaemia Score			*p*-Trend	Dietary Atherogenic Score			*p*-Trend
		Q2	Q3	Q4		Q2	Q3	Q4	
**All-Cause Mortality**	**Model 1**	1.13 (0.55–1.37)	1.12 (1.08–1.19)	1.30 (1.12–1.45)	0.029	1.02 (0.81–1.27)	1.30 (1.19–1.45)	1.40 (1.09–1.81)	0.018
	**Model 2**	1.29 (0.87–1.96)	1.09 (1.05–1.15)	1.17 (1.07–1.30)	0.034	1.13 (0.62–1.75)	1.09 (1.05–1.15)	1.25 (1.20–1.30)	0.042
**Cardiovascular Disease Mortality**	**Model 1**	1.11 (1.04–1.17)	1.16 (1.12–1.20)	1.45 (1.12–1.89)	<0.001	1.16 (1.05–1.28)	1.34 (1.19–1.55)	1.55 (1.45–1.62)	<0.001
	**Model 2**	1.39 (0.84–2.32)	1.04(1.02–1.07)	1.36 (1.21–1.59)	<0.001	1.08 (1.02–1.13)	1.28 (1.05–1.54)	1.40 (1.10–1.79)	<0.001
**Cancer Mortality**	**Model 1**	1.09 (0.96–1.14)	0.90 (0.78–1.09)	1.11 (1.04–1.18)	0.248	1.20 (0.88–1.62)	1.17 (0.90–1.82)	1.15 (1.08–1.17)	0.468
	**Model 2**	1.01 (0.96–1.08)	0.94 (0.81–1.04)	1.06 (1.01–1.14)	0.302	1.00 (0.78–1.28)	1.06 (0.72–1.55)	1.11 (1.04–1.19)	0.327

Model 1: Adjusted for age, gender, race, education, marital status, poverty to income ratio, total energy intake, physical activity, smoking and alcohol consumption.; Model 2: Adjusted for age, gender, race, education, marital status, poverty to income ratio, total energy intake, physical activity, smoking, alcohol consumption, body mass index, hypertension and type 2 diabetes mellitus.; First quartile is always the reference group.

## References

[1] Beaglehole R., Bonita R., Horton R., Adams C., Alleyne G., Asaria P., Baugh V., Bekedam H., Billo N., Casswell S. (2011). Priority actions for the non-communicable disease crisis. Lancet.

[2] Melaku Y.A., Renzaho A., Gill T.K., Taylor A.W., Dal Grande E., de Courten B., Baye E., Gonzalez-Chica D., Hyppönen E., Shi Z. (2018). Burden and trend of diet-related non-communicable diseases in Australia and comparison with 34 OECD countries, 1990–2015: Findings from the Global Burden of Disease Study 2015. Eur. J. Nutr..

[3] http://www.who.int/news-room/fact-sheets/detail/noncommunicable-diseases.

[4] Kurotani K., Akter S., Kashino I., Goto A., Mizoue T., Noda M., Sasazuki S., Sawada N., Tsugane S. (2016). Quality of diet and mortality among Japanese men and women: Japan Public Health Center based prospective study. BMJ.

[5] Jannasch F., Kröger J., Schulze M.B. (2017). Dietary Patterns and Type 2 Diabetes: A Systematic Literature Review and Meta-Analysis of Prospective Studies–3. J. Nutr..

[6] Perez-Martinez P., Mikhailidis D.P., Athyros V.G., Bullo M., Couture P., Covas M.I., de Koning L., Delgado-Lista J., Diaz-Lopez A., Drevon C.A. (2017). Lifestyle recommendations for the prevention and management of metabolic syndrome: An international panel recommendation. Nutr. Rev..

[7] Estruch R., Ros E., Salas-Salvadó J., Covas M.-I., Corella D., Arós F., Gómez-Gracia E., Ruiz-Gutiérrez V., Fiol M., Lapetra J. (2013). Primary prevention of cardiovascular disease with a Mediterranean diet. N. Engl. J. Med..

[8] Ruiz-Canela M., Estruch R., Corella D., Salas-Salvadó J., Martínez-González M.A. (2014). Association of Mediterranean diet with peripheral artery disease: The PREDIMED randomized trial. JAMA.

[9] Toledo E., Salas-Salvadó J., Donat-Vargas C., Buil-Cosiales P., Estruch R., Ros E., Corella D., Fitó M., Hu F.B., Arós F. (2015). Mediterranean diet and invasive breast cancer risk among women at high cardiovascular risk in the PREDIMED trial: A randomized clinical trial. JAMA Intern. Med..

[10] Schulze M.B., Martínez-González M.A., Fung T.T., Lichtenstein A.H., Forouhi N.G. (2018). Food based dietary patterns and chronic disease prevention. BMJ.

[11] Kushiyama A., Nakatsu Y., Matsunaga Y., Yamamotoya T., Mori K., Ueda K., Inoue Y., Sakoda H., Fujishiro M., Ono H. (2016). Role of Uric Acid Metabolism-Related Inflammation in the Pathogenesis of Metabolic Syndrome Components Such as Atherosclerosis and Nonalcoholic Steatohepatitis. Mediat. Inflamm..

[12] Li Q., Zhou Y., Dong K., Wang A., Yang X., Zhang C., Zhu Y., Wu S., Zhao X. (2015). The Association between Serum Uric Acid Levels and the Prevalence of Vulnerable Atherosclerotic Carotid Plaque: A Cross-sectional Study. Sci. Rep..

[13] Wannamethee S.G., Papacosta O., Lennon L., Whincup P.H. (2018). Serum uric acid as a potential marker for heart failure risk in men on antihypertensive treatment: The British Regional Heart Study. Int. J. Cardiol..

[14] Dovell F., Boffetta P. (2018). Serum uric acid and cancer mortality and incidence: A systematic review and meta-analysis. Eur. J. Cancer Prev..

[15] Huang H., Huang B., Li Y., Huang Y., Li J., Yao H., Jing X., Chen J., Wang J. (2014). Uric acid and risk of heart failure: A systematic review and meta-analysis. Eur. J. Heart Fail..

[16] Zhao G., Huang L., Song M., Song Y. (2013). Baseline serum uric acid level as a predictor of cardiovascular disease related mortality and all-cause mortality: A meta-analysis of prospective studies. Atherosclerosis.

[17] Juraschek S.P., Gelber A.C., Choi H.K., Appel L.J., Miller E.R. (2016). Effects of the Dietary Approaches to Stop Hypertension (DASH) Diet and Sodium Intake on Serum Uric Acid. Arthritis Rheumatol..

[18] Tang O., Miller E.R., Gelber A.C., Choi H.K., Appel L.J., Juraschek S.P. (2017). DASH diet and change in serum uric acid over time. Clin. Rheumatol..

[19] Juraschek S.P., White K., Tang O., Yeh H.-C., Cooper L.A., Miller E.R. (2018). Effects of a Dietary Approach to Stop Hypertension (DASH) Diet Intervention on Serum Uric Acid in African Americans With Hypertension. Arthritis Care Res..

[20] Sniderman A.D., Islam S., McQueen M., Pencina M., Furberg C.D., Thanassoulis G., Yusuf S. (2016). Age and Cardiovascular Risk Attributable to Apolipoprotein B, Low-Density Lipoprotein Cholesterol or Non-High-Density Lipoprotein Cholesterol. J. Am. Heart Assoc. Cardiovasc. Cerebrovasc. Dis..

[21] Boren J., Williams K.J. (2016). The central role of arterial retention of cholesterol-rich apolipoprotein-B-containing lipoproteins in the pathogenesis of atherosclerosis: A triumph of simplicity. Curr. Opin. Lipidol..

[22] Lamantia V., Sniderman A., Faraj M. (2016). Nutritional management of hyperapoB. Nutr. Res. Rev..

[23] Poggio R., Elorriaga N., Gutierrez L., Irazola V., Rubinstein A., Danaei G. (2017). Associations between dietary patterns and serum lipids, apo and C-reactive protein in an adult population: Evidence from a multi-city cohort in South America. Br. J. Nutr..

[24] Steinberg H.O., Stentz F.B., Shankar N.K. (2018). Effects of a High-Carb vs. High-Fat Meal on Glycemia, Insulin, Interleukin-6, TNF-Alpha, and Apo-B. Diabetes.

[25] Mazidi M., Wong N.D., Katsiki N., Mikhailidis D.P., Banach M. (2017). Dietary patterns, plasma vitamins and Trans fatty acids are associated with peripheral artery disease. Lipids Health Dis..

[26] Hu F.B. (2002). Dietary pattern analysis: A new direction in nutritional epidemiology. Curr. Opin. Lipidol..

[27] Newby P., Tucker K.L. (2004). Empirically derived eating patterns using factor or cluster analysis: A review. Nutr. Rev..

[28] Movassagh E.Z., Vatanparast H. (2017). Current Evidence on the Association of Dietary Patterns and Bone Health: A Scoping Review. Adv. Nutr..

[29] Mazidi M., Kengne A.P. (2017). Nutrient patterns and their relationship with general and central obesity in US adults. Eur. J. Clin. Investig..

[30] Mazidi M., Pennathur S., Afshinnia F. (2017). Link of dietary patterns with metabolic syndrome: Analysis of the National Health and Nutrition Examination Survey. Nutr. Diabetes.

[31] Slattery M.L. (2008). Defining dietary consumption: Is the sum greater than its parts?. Am. J. Clin. Nutr..

[32] Mazidi M., Katsiki N., Mikhalidis D.P., Banach M. (2018). The link between insulin resistance parameters and serum uric acid is mediated by adiposity. Atherosclerosis.

[33] Major T.J., Topless R.K., Dalbeth N., Merriman T.R. (2018). Evaluation of the diet wide contribution to serum urate levels: Meta-analysis of population based cohorts. BMJ.

[34] Furtado J.D., Campos H., Appel L.J., Miller E.R., Laranjo N., Carey V.J., Sacks F.M. (2008). Effect of protein, unsaturated fat, and carbohydrate intakes on plasma apolipoprotein B and VLDL and LDL containing apolipoprotein C-III: Results from the OmniHeart Trial. Am. J. Clin. Nutr..

[35] Frondelius K., Borg M., Ericson U., Borne Y., Melander O., Sonestedt E. (2017). Lifestyle and Dietary Determinants of Serum Apolipoprotein A1 and Apolipoprotein B Concentrations: Cross-Sectional Analyses within a Swedish Cohort of 24,984 Individuals. Nutrients.

[36] National Center for Health Statistics Office of Analysis and Epidemiology P-uNILMf, 2007. Maryland, Hyattsville. National Center for Health Statistics. Office of Analysis and Epidemiology, Public-use NHANES III Linked Mortality File, 2007. Maryland, Hyattsville. http://www.cdc.gov/nchs/r&d/nchs_datalinkage/nhanes3_data_linkage_mortality_activities.htm.

[37] Tighe P., Duthie G., Vaughan N., Brittenden J., Simpson W.G., Duthie S., Mutch W., Wahle K., Horgan G., Thies F. (2010). Effect of increased consumption of whole-grain foods on blood pressure and other cardiovascular risk markers in healthy middle-aged persons: A randomized controlled trial. Am. J. Clin. Nutr..

[38] Mazidi M., Kengne A.P. (2019). Higher adherence to plant-based diets are associated with lower likelihood of fatty liver. Clin. Nutr..

[39] Mohsen Mazidi E.D.M., Banach M. (2017). The association of telomere length and serum 25-hydroxyvitamin D levels in US adults: The National Health and Nutrition Examination Survey. Arch. Med. Sci..

[40] National Center for Health Statistics. http://www.cdc.gov/nchs/nhanes.html.

[41] Nwankwo T., Yoon S.S., Burt V., Gu Q. (2013). Hypertension among adults in the United States: National Health and Nutrition Examination Survey, 2011–2012. Nchs Data Brief.

[42] (1997). Report of the Expert Committee on the Diagnosis and Classification of Diabetes Mellitus. Diabetes Care.

[43] Ahluwalia N., Andreeva V.A., Kesse-Guyot E., Hercberg S. (2013). Dietary patterns, inflammation and the metabolic syndrome. Diabetes Metab.

[44] Ahluwalia N., Dwyer J., Terry A., Moshfegh A., Johnson C. (2016). Update on NHANES Dietary Data: Focus on Collection, Release, Analytical Considerations, and Uses to Inform Public Policy. Adv. Nutr..

[45] Statistics NCfH Analytic and Reporting Guidelines. http://www.cdc.gov/nchs/data/nhanes/nhanes0304/nhanesanalyticguidelinesdec2005.pdf.

[46] Babio N., Martínez-González M.A., Estruch R., Wärnberg J., Recondo J., Ortega-Calvo M., Serra-Majem L., Corella D., Fitó M., Ros E. (2015). Associations between serum uric acid concentrations and metabolic syndrome and its components in the PREDIMED study. Nutr. Metab. Cardiovasc. Dis..

[47] Keenan T., Zhao W., Rasheed A., Ho W.K., Malik R., Felix J.F., Young R., Shah N., Samuel M., Sheikh N. (2016). Causal Assessment of Serum Urate Levels in Cardiometabolic Diseases Through a Mendelian Randomization Study. J. Am. Coll Cardiol.

[48] Fang J., Alderman M.H. (2000). Serum uric acid and cardiovascular mortality the NHANES I epidemiologic follow-up study, 1971–1992. National Health and Nutrition Examination Survey. JAMA.

[49] Choi Y.-J., Yoon Y., Lee K.-Y., Hien T.T., Kang K.W., Kim K.-C., Lee J., Lee M.-Y., Lee S.M., Kang D.-H. (2014). Uric acid induces endothelial dysfunction by vascular insulin resistance associated with the impairment of nitric oxide synthesis. FASEB J..

[50] Ho W.-J., Tsai W.-P., Yu K.-H., Tsay P.-K., Wang C.-L., Hsu T.-S., Kuo C.-T. (2010). Association between endothelial dysfunction and hyperuricaemia. Rheumatology.

[51] Kontogianni M.D., Chrysohoou C., Panagiotakos D.B., Tsetsekou E., Zeimbekis A., Pitsavos C., Stefanadis C. (2012). Adherence to the Mediterranean diet and serum uric acid: The ATTICA study. Scand. J. Rheumatol..

[52] Cho S.K., Chang Y., Kim I., Ryu S. (2018). U-Shaped Association Between Serum Uric Acid Level and Risk of Mortality. Arthritis Rheumatol..

[53] Beberashvili I., Sinuani I., Azar A., Shapiro G., Feldman L., Stav K., Sandbank J., Averbukh Z. (2015). Serum uric acid as a clinically useful nutritional marker and predictor of outcome in maintenance hemodialysis patients. Nutrition.

[54] Johnson R.J., Kang D.H., Feig D., Kivlighn S., Kanellis J., Watanabe S., Tuttle K.R., Rodriguez-Iturbe B., Herrera-Acosta J., Mazzali M. (2003). Is there a pathogenetic role for uric acid in hypertension and cardiovascular and renal disease?. Hypertension.

[55] Richard C., Couture P., Ooi E.M., Tremblay A.J., Desroches S., Charest A., Lichtenstein A.H., Lamarche B. (2014). Effect of Mediterranean diet with and without weight loss on apolipoprotein B100 metabolism in men with metabolic syndrome. Arter. Thromb. Vasc. Biol..

[56] Gesteiro E., Bastida S., Rodríguez Bernal B., Sánchez-Muniz F.J. (2015). Adherence to Mediterranean diet during pregnancy and serum lipid, lipoprotein and homocysteine concentrations at birth. Eur. J. Nutr..

[57] Srichaikul K., Ireland C., Kendall C., Sievenpiper J., Lamarche B., Jenkins D. (2015). Effect of Low Glycemic Index Diet On Apolipoprotein B and Ldl Particle Size. FASEB J..

